# Effects of Intravenous Dextrose Timing on Postoperative Nausea, Vomiting and Anxiety

**DOI:** 10.4274/TJAR.2025.252018

**Published:** 2025-10-14

**Authors:** Yusuf Özgüner, Savaş Altınsoy, İsmet Uluhan, Funda Atar, Derya Özkan, Jülide Ergil

**Affiliations:** 1 University of Health Sciences Türkiye Ankara Etlik City Hospital, Clinic of Anaesthesiology and Reanimation, Ankara, Türkiye

**Keywords:** Anaesthesia, anxiety, laparoscopic cholecystectomy, postoperative nausea and vomiting

## Abstract

**Objective:**

Postoperative nausea and vomiting (PONV) is a significant issue encountered in surgical patients. This study aims to investigate the effects of dextrose infusion timing on PONV incidence.

**Methods:**

Ninety patients undergoing laparoscopic cholecystectomy were included in this randomized controlled trial. Patients were assigned to one of three equal groups. In Group I, patients received an infusion of 400 mL of 0.9% saline 2 hours before surgery. In Group D, patients received 400 mL of 5% dextrose at the same infusion rate. Both Groups I and D received 0.9% saline at 10 mL kg^-1^ h^-1^ during the intraoperative period. In Group DD, patients received 200 mL of 5% dextrose preoperatively and 200 mL intraoperatively. To ensure the total maintenance fluid volume was the same as in the other groups, an infusion of 0.9% saline was administered along with the 200 mL dextrose. The primary outcome in our study was PONV incidence. Secondary outcomes were postoperative pain and anxiety levels.

**Results:**

Postoperative PONV incidence, antiemetic consumption, and anxiety levels were lowest in Group DD, while they were highest in Group I (*P* < 0.05).

**Conclusion:**

In this study, we found that dextrose infusion reduced the incidence of PONV, antiemetic consumption, and anxiety levels. We observed that administering the same volumes of dextrose in divided doses during the preoperative and intraoperative periods reduced the incidence of PONV and improved anxiety scores compared to sole preoperative dextrose infusion.

**Main Points**

• Dextrose infusion is effective in postoperative nausea and vomiting (PONV) prophylaxis.

• There is a relationship between blood glucose levels and PONV.

• In addition to reducing the incidence of PONV, dextrose infusion also decreases anxiety levels.

• Administering dextrose in the same doses preoperatively and intraoperatively results in lower incidence of PONV and anxiety levels compared to administering dextrose alone preoperatively.

## Introduction

Pain, postoperative nausea and vomiting (PONV) are significant issues encountered in surgical patients. The incidence of PONV ranges from 30% to 80%.^1, 2^ Risk factors for PONV include female gender, being under age 50, non-smoking status, motion sickness, laparoscopic cholecystectomy, and gynaecological surgeries.^3^ In laparoscopic surgeries, abdominal gas insufflation is thought to increase the risk of PONV by stimulating mechanoreceptors in the intestines, leading to serotonin release and the activation of 5-HT3 receptors.^4, 5^ Additionally, factors such as anxiety and stress have been reported to cause nausea and vomiting.^6^

Untreated PONV can lead to dehydration, electrolyte imbalance, aspiration, and bleeding. This results in reduced patient satisfaction, prolonged hospital stays, and increased medical care costs.^3, 7^ Various pharmacological agents, such as serotonin 5-HT3 receptor antagonists, dopamine receptor antagonists (metoclopramide), antihistamines, and steroids, are used to treat PONV.^5, 8^ However, these pharmacological agents are associated with side effects, such as extrapyramidal symptoms, sedation, and hyperglycaemia. Many studies have investigated the effectiveness of perioperative fluid therapy in PONV prophylaxis. The infusion of perioperative dextrose at different times and doses reduces the incidence of PONV and the use of antiemetic drugs.^9, 10, 11^

The primary aim of our study was to investigate the hypothesis that patients receiving both preoperative and intraoperative dextrose infusions would have a lower incidence of PONV than those receiving only preoperative saline or dextrose infusions. Our secondary aim was to examine the differences in pain scores and anxiety levels between the groups.

## Methods

### Trial Design

This single-center randomized controlled study complied with the ethical standards of the Helsinki Declaration-2013. Ethics committee approval was obtained from the University of Health Sciences Türkiye, Dışkapı Yıldırım Beyazıt Training and Research Hospital Clinical Research Ethics Committee (approval no.: 142/07, date: 18.07.2022) and registration with ClinicalTrials.gov were obtained (registration number: NCT05961722-13.07.2023). Written informed consent was obtained from all the participants.

### Participants

Female and male patients undergoing laparoscopic cholecystectomy were included in the study. Patients over the age of 18, classified as American Society of Anesthesiologists I-II, who agreed to participate, were included. Patients with a history of PONV or motion sickness, those with diabetes mellitus or hypothyroidism, pregnant women, and individuals receiving opioids, chemotherapy, steroids, or antiemetic treatment, were excluded from the study.

### Interventions

Patients were randomly assigned to one of three groups using sealed envelopes. An anaesthetist, blinded to patient treatment conditions, followed the patients postoperatively. In Group I, patients received an infusion of 400 mL of 0.9% saline over 30 minutes, administered 2 hours before surgery. In Group D, patients received 400 mL of 5% dextrose over 30 minutes, administered 2 hours before surgery. In Group DD, patients received 200 mL of 5% dextrose over 30 minutes, administered 2 hours before surgery. Both Groups I and D received 0.9% saline at 10 mL kg^-1^ h^-1^ during the intraoperative period. In Group DD, patients received 200 ml intraoperatively. To ensure the total maintenance fluid volume was the same as in the other groups, an infusion of 0.9% saline was administered along with the 200 mL dextrose.

### Standard Anaesthesia Protocol

For all three groups, anaesthesia induction included 2 mg kg^-1^ of propofol, 1 mg kg^-1^ of lidocaine, 1 μg kg^-1^ of fentanyl, and 0.6 mg kg^-1^ of rocuronium. After anaesthesia induction, patients underwent endotracheal intubation. Anaesthesia maintenance in both groups involved the administration of 0.8-1.2 minimum alveolar concentration sevoflurane and 0.05-0.2 μg kg^-1^ min^-1^ remifentanil. Patients in both groups were monitored during surgery using volume-controlled ventilation mode with 50% oxygen-50% air, a tidal volume of 6 mL kg^-1^, and a respiratory rate of 12 breaths per minute. Patient monitoring in both groups included pulse oximetry (SpO_2_), end-tidal carbon dioxide, heart rate, non-invasive blood pressure, Bispectral index, temperature, and urine output. Blood glucose measurements were taken using a glucometer before preoperative fluid infusion, after intraoperative anaesthesia induction, and when the patient was transferred to the postoperative recovery room. All patients received intravenous 100 mg tramadol and 50 mg dexketoprofen for analgesia at the end of surgery, and 4 mg ondansetron as an antiemetic. At the end of the operation, the reversal of the neuromuscular blockade was achieved using 50 μg kg^-1^ neostigmine plus 10 μg kg^-1^ atropine. Patients were extubated at the end of surgery and transferred to the recovery room. In the first 24 hours postoperatively, all patients routinely received 1 mg kg^-1^ intravenous tramadol and 2 g intravenous paracetamol administered twice for analgesia.

### Outcomes

The primary outcome in our study was the 24-hour postoperative incidence of PONV. Secondary outcomes were postoperative pain and anxiety levels.

### Evaluation of PONV

PONV score and antiemetic requirements were assessed using the Verbal Descriptive Scale (VDS) at 0, 2, 4, 8, 12, and 24 hours. Patients with VDS of 2 and 3 were treated with 4 mg IV ondansetron. VDS:^12^

• 0= no PONV: patient reports no nausea and has had no emesis episodes;

• 1= mild PONV: patient reports nausea but declines antiemetic treatment;

• 2= moderate PONV: patient reports nausea and accepts antiemetic treatment;

• 3= severe PONV: nausea with any emesis episode (retching or vomiting).

### Evaluation of Pain

Pain levels of the patients were assessed using the numeric rating scale (NRS), which ranges from 0 to 10. A score of 0 indicates no pain, while a score of 10 represents the worst possible pain. Pain was assessed using the NRS at 0, 2, 4, 8, 12, and 24 hours. Patients with an NRS score above 4 received 50 mg of dexketoprofen as rescue analgesia.

### Evaluation of Anxiety

The State-Trait Anxiety Inventory (STAI) scale is frequently used to assess anxiety. STAI-1 is used to measure state anxiety, reflecting the individual’s current level of anxiety, while STAI-2 assesses trait anxiety, indicating the individual’s general tendency toward anxiety. The validity and reliability of the scale were established by Oner and LeCompte^13^ in Türkiye. High scores indicate high anxiety levels, and low scores indicate low anxiety levels. The scale contains four scores ranging from “never” to “completely”.^13, 14^ Anxiety levels of all patients were evaluated preoperatively using the STAI 1 and 2 anxiety scales before intravenous fluid infusion. The STAI 1 scale was reapplied at 4-6 hours postoperatively.^15^

### Statistical Analysis

The sample size was determined assuming an expected prevalence of PONV of 60%, and 29 patients per group were found to be adequate for detecting an absolute 35% reduction in PONV (α=0.05, 1-ß=0.80). According to the preliminary study, the total sample size required was calculated as 87 patients. Taking potential dropouts into account, 96 patients were included in the study. SPSS 21.0 software was used for statistical analysis. The chi-square test (for categorical variables), One-Way ANOVA (for continuous variables with normal distribution), and Kruskal-Wallis test (for continuous variables with non-normal distribution) were employed in this study. A *P *value of < 0.05 was considered statistically significant. The Tukey test was used for multiple comparisons between the groups.

## Results

**Figure 1:**
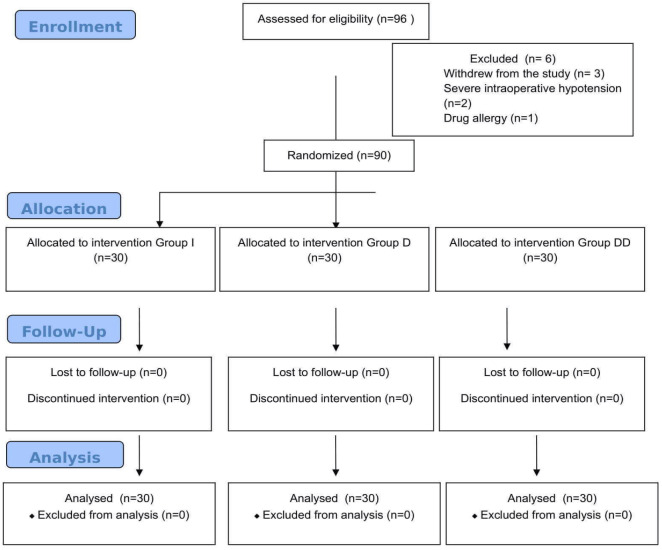
CONSORT diagram of the study.

Ninety-six patients undergoing laparoscopic cholecystectomy were included in the study. From the study, Two patients who experienced severe intraoperative hypotension, three patients who withdrew from the study, and one patient with a drug allergy were excluded. Consequently, a total of 90 patients were included in the final analysis (Figure 1).

The demographic and clinical characteristics (age, gender, ASA classification, body mass index, chronic diseases, average duration of surgery, total intravenous fluid volumes) were similar across the groups (Table 1).

PONV was observed in 19 patients (63.3%) in Group I, 10 patients (33.3%) in Group D, and 3 patients (10%) in Group DD. Postoperative rescue antiemetics were used in 16 patients in Group I, in 8 patients in Group D, and in 2 patients in Group DD. The highest incidence of PONV and postoperative antiemetic use was in Group I, while the lowest was in Group DD (*P* < 0.05) (Table 2).

NRS values and rescue analgesic consumption were similar across all groups at all postoperative time points (*P* > 0.05) (Table 3).

Preoperative blood glucose concentrations were comparable. Intraoperatively, Group I had a mean glucose level of 96.8±5.01, Group D had 149.23±6.41, and Group DD had 130.77±7.72. Postoperatively, Group I had 107.77±6.05, Group D had 151±7.11, and Group DD had 152.93±6.01. The highest intraoperative glucose levels were observed in Group D, while the lowest were in Group I. Postoperatively, there was no significant difference between Group D and Group DD, but Group I had the lowest levels (Table 4).

Preoperatively, STAI-1 and STAI-2 anxiety scores were similar among the three groups (*P* > 0.05). Postoperatively, the highest average STAI-1 score was observed in Group I, while the lowest was in Group DD (*P* < 0.05) (Table 4).

## Discussion

In our study investigating the effects of dextrose versus saline infusions on PONV during preoperative and intraoperative periods, we found that the group receiving saline infusion alone had a higher incidence of PONV, increased antiemetic consumption, and higher anxiety scores. In contrast, patients who received dextrose infusions during both the preoperative and intraoperative periods exhibited lower incidences of PONV, reduced need for antiemetic medications, and lower anxiety levels than the other two groups.

Studies investigating the effects of fluid infusion therapy on PONV have reported that the effectiveness of crystalloid and colloid fluids is limited.^16, 17^ However, other studies have indicated that a dextrose infusion administered during the postoperative period reduces the incidence of PONV.^18, 19^ Mishra et al.^20^ reported that intraoperatively administered dextrose infusions decreased the incidence of PONV. Salman et al.^10^ compared preoperative and intraoperative dextrose infusions and found that both approaches were effective in preventing PONV. However, preoperative dextrose infusions have resulted in a lower incidence of PONV. Consistent with the literature, we observed a reduced incidence of PONV with dextrose infusions. The relationship between dextrose infusions and PONV is not clearly understood. Hyperglycaemia may lead to reduced gastric acid secretion, which decreases gastric contractions and alleviates nausea.^21^ Additionally, dextrose infusions are believed to provide caloric supplementation, reducing catabolism and insulin resistance, thereby decreasing the risk of PONV.^20, 22^ In line with these findings, we hypothesised that the administration of dextrose infusions would be associated with a reduced incidence of PONV in comparison to those who did not.

In the literature, studies investigating the effect of dextrose infusion on PONV have employed varying dosages, timings, and durations of administration. Furthermore, there is currently no consensus regarding the optimal dextrose infusion protocol. In this study, when comparing two groups that received dextrose infusions at the same volumes, we found that only the patients who received dextrose infusions in the preoperative period had a higher incidence of PONV. Feldbauer et al.^23^ reported that infusing glucose at the same dosage for longer durations resulted in fewer fluctuations in blood glucose levels. We observed a rapid increase in intraoperative blood glucose levels in patients in Group D, compared to the preoperative period, while in Group DD, blood glucose levels increased more slowly and gradually. We believe that administering the same volumes of dextrose infusion divided between the preoperative and intraoperative periods resulted in fewer fluctuations in patients’ blood glucose levels than infusions administered solely during the preoperative period, potentially contributed to the reduced incidence of PONV. Additionally, we think that continued caloric intake through dextrose infusions during the intraoperative period helped decrease catabolism, thereby contributing to this outcome. Therefore, we consider the timing and rate of dextrose administration to be critical factors in maintaining metabolic stability and promoting postoperative recovery.

The literature has identified the causes of postoperative anxiety. Factors such as gender, age, type of anaesthesia (general or regional), educational status, and type of surgery significantly affect postoperative anxiety. Furthermore, postoperative anxiety has the potential to adversely influence multiple clinical outcomes, such as pain levels and patient comfort.^24, 25^ We observed that the factors known to influence anxiety were similar among the patient groups. Additionally, a relationship between preoperative fasting duration and postoperative anxiety has been established. Hausel et al.^26^ reported in their studies involving patients undergoing abdominal surgery that the preoperative intake of oral carbohydrate-containing solutions reduced anxiety levels. Mousavie et al.^27^ also indicated that preoperative oral and intravenous dextrose replacements positively impacted patients’ emotional states. Consistent with the literature, our study found that patients receiving dextrose infusions had lower postoperative anxiety levels. Patients receiving both preoperative and intraoperative dextrose infusions exhibited lower anxiety scores than those receiving only preoperative dextrose infusions. Increased blood glucose levels are known to elevate plasma cholecystokinin levels. Cholecystokinin has been reported to play a role in the regulation of pain and anxiety.^11, 28^ Therefore, we believe that the lower anxiety levels observed in patients receiving dextrose infusions may be attributed to differences in cholecystokinin levels. We also propose that the lower anxiety scores in Group DD, than in Group D may be due to the division of dextrose infusion into preoperative and intraoperative periods with the same total volume. Furthermore, we believe that an ongoing dextrose infusion during the intraoperative period helps to reduce catabolism and the effects of surgical stress. Nonetheless, the potential benefits of dextrose infusion should be balanced with careful monitoring of blood glucose levels to prevent hyperglycemia-related complications. Future research focusing on the optimization of infusion timing and dosage could further enhance patient outcomes and provide clearer guidelines for clinical practice.

### Study Limitations

Our study has certain limitations. First, not monitoring hormone levels such as insulin, aside from blood glucose levels, was a limitation. Second, we were unable to evaluate the long-term effects and hospital stay duration as we did not follow patients beyond the first 24 hours postoperatively.

## Conclusion

We found that dextrose infusion reduced the incidence of PONV, antiemetic consumption, and anxiety levels. We observed that administering the same volumes of dextrose in divided doses during the preoperative and intraoperative periods positively affected the incidence of PONV and anxiety scores compared to sole preoperative dextrose infusion. Therefore, we believe that dextrose infusion, as a low-cost method, is an effective strategy for PONV prophylaxis in surgical patients during both the preoperative and intraoperative periods.

## Ethics

**Ethics Committee Approval:** Ethics committee approval was obtained from the University of Health Sciences Türkiye, Dışkapı Yıldırım Beyazıt Training and Research Hospital Clinical Research Ethics Committee (approval no.: 142/07, date: 18.07.2022).

**Informed Consent:** Written informed consent was obtained from all the participants.

## Figures and Tables

**Table 1. Demographic and Clinical Characteristics of the Study Patients:** 

-	**Group I** **n = 30**	**Group D** **n = 30**	**Group DD** **n = 30**	***P* value**
Age (year)	48.87±13.48	48.63±7.25	48.13±8.91	0.961
Sex (n) Female/Male	15/15	14/16	14/16	0.956
BMI (kg m^2-1^)	27.59±3.05	27.31±2.77	27.62±3.04	0.902
ASA score (1/2) (n)	13/17	14/16	11/19	0.727
Duration of surgery (minute)	53.5±7.31	56.27±7.46	57.13±9.39	0.200
Volume of fluid administered (mL)	1076.66±127.74	1099.66±142.5	1094.66±122.77	0.776
**Comorbidities** Hypertension Smoking Asthma	- 10 8 3	- 9 7 2	- 10 7 2	- 0.950 0.943 0.856

Values ​​are presented as mean ± standard deviation and numbers. The chi-square test was used for categorical variables, One-Way ANOVA for continuous variables with normal distribution, and the Tukey test for multiple group comparisons.

n, number; BMI, body mass index; ASA, American Society of Anesthesiologists.

**Table 2. PONV Incidence and Severity:** 

-	**Group I** **n = 30**	**Group D** **n = 30**	**Group DD** **n = 30**	***P *value**
**PONV (n, %)**	19 (63.3)	10 (33.3)	3 (10)	<0.001*
**PONV score (n)** 0/1/2/3 0^th^ hour 2^nd^ hour 4^th^ hour 8^th^ hour 12^th^ hour 24^th^ hour	- - 26/1/1/2 9/12/8/1 15/13/2/0 24/3/2/1 26/1/1/2 29/1/0/0	- - 28/1/1/0 20/6/3/1 24/6/0/0 25/2/2/1 28/1/1/0 30/0/0/0	- - 30/0/0/0 28/1/0/1 29/1/0/0 27/2/1/0 30/0/0/0 30/0/0/0	- - <0.001* <0.001* <0.001* 0.931 0.392 0.364
Required antiemetic (n)	16	8	2	<0.001*

Values ​​are presented as numbers. n, number/percentages. P < 0.05 was considered significant. *There were significant differences among the three groups. The chi-square test was used for categorical variables.

PONV, postoperative nausea and vomiting.

**Table 3. Pain Scores (NRS) and Rescue Analgesic Consumption:** 

-	**Group I** **n = 30**	**Group D** **n = 30**	**Group DD** **n = 30**	***P *value**
NRS 0^th^	3 (5)	3 (4)	3 (5)	0.464
NRS 2^nd^	4 (4)	4 (3)	4 (3)	0.481
NRS 4^th^	3 (4)	3 (3)	2 (4)	0.834
NRS 8^th^	2.5 (4)	3 (3)	3 (4)	0.987
NRS 12^th^	3 (3)	3 (3)	2 (3)	0.300
NRS 24^th^	2 (3)	2 (2)	2 (2)	0.773
Rescue analgesic (n)	6	4	4	0.713

Values ​​are given as median (range) and numbers. *P *< 0.05 was considered significant. The chi-square test was used for categorical variables, while the Kruskal-Wallis test was applied to continuous variables that did not follow a normal distribution.

NRS, numeric rating score.

**Table 4. Anxiety Score and Blood Glucose Levels:** 

-	**Group I** **n = 30**	**Group D** **n = 30**	**Group DD** **n = 30**	***P* value**
Preoperative STAI 1 score	49.23±6.11	49.63±6.62	49.73±6.28	0.949
Preoperative STAI 2 score	42.7±8.45	41.57±6.98	42.83±8.16	0.792
Postoperative STAI 1 score	45.27±6.24	40.87±5.88	36.93±6.5	<0.001*
Preoperative blood glucose (mg dL^-1^)	87.67±4.7	88.50±5.32	87.47±6.95	0.761
Intraoperative blood glucose (mg dL^-1^)	96.8±5.01	149.23±6.41	130.77±7.72	<0.001*
Postoperative blood glucose (mg dL^-1^)	107.77±6.05	151±7.11	152.93±6.01	**<0.001#**

Values ​​are given as mean ± standard deviation. *There were significant differences among the three groups. #There is a difference between Group I and the other two groups. One-Way ANOVA was used for continuous variables with normal distribution, and the Tukey test was applied for multiple group comparisons.

STAI, State-Trait Anxiety Inventory.

## References

[1] Gan TJ (2002). Postoperative nausea and vomiting--can it be eliminated?. JAMA.

[2] Carlisle JB, Stevenson CA (2006). Drugs for preventing postoperative nausea and vomiting.. Cochrane Database Syst Rev.

[3] Apfel CC, Kranke P, Eberhart LH, Roos A, Roewer N (2002). Comparison of predictive models for postoperative nausea and vomiting.. Br J Anaesth.

[4] Liao B, Liao W, Wu X (2024). Analysis of influencing factors and construction of prediction model for postoperative nausea and vomiting in patients undergoing laparoscopic sleeve gastrectomy: a single-center retrospective cohort study.. BMC Anesthesiol.

[5] Apfel CC, Korttila K, Abdalla M (2004). A factorial trial of six interventions for the prevention of postoperative nausea and vomiting.. N Engl J Med.

[6] Pasyar N, Rambod M, Zahedi F, Ramzi M (2022). Pain, fatigue, nausea, and vomiting as the predictors of anxiety in patients undergoing hematopoietic stem cell transplantation: a prospective cohort study.. Support Care Cancer.

[7] Stadler M, Bardiau F, Seidel L, Albert A, Boogaerts JG (2003). Difference in risk factors for postoperative nausea and vomiting.. Anesthesiology.

[8] Gan TJ, Diemunsch P, Habib AS (2014). Consensus guidelines for the management of postoperative nausea and vomiting.. Anesth Analg.

[9] D’Souza RS, Mercogliano C, Ojukwu E (2018). Effects of prophylactic anticholinergic medications to decrease extrapyramidal side effects in patients taking acute antiemetic drugs: a systematic review and meta-analysis.. Emerg Med J.

[10] Salman N, Aykut A, Sabuncu Ü, Şaylan A, Yağar S, Şekerci S (2020). Dextrose administration may reduce the incidence of postoperative nausea and vomiting after laparoscopic cholecystectomy: a double blind randomized controlled trial.. Minerva Anestesiol.

[11] Deng R, Huang G, Liu W, Liu X (2021). Effects of perioperative dextrose infusion on preventing postoperative nausea and vomiting in patients undergoing laparoscopic surgery: a meta-analysis of randomized controlled trials.. J Int Med Res.

[12] Boogaerts JG, Vanacker E, Seidel L, Albert A, Bardiau FM (2000). Assessment of postoperative nausea using a visual analogue scale.. Acta Anaesthesiol Scand.

[13] Oner N, LeCompte A. Durumluk − Surekli Kaygı Envanteri El Kitabı. 1. Baskı Istanbul: Boğaziçi Üniversitesi Yayını; 1983. s. 1-26..

[14] Topal Hançer A (2023). Prevalence and factors associated with surgery anxiety in hospitalized patients: a point-prevalence study.. Ir J Med Sci.

[15] Akelma FK, Altınsoy S, Nalbant B, Özkan D, Ergil J (2024). Comparison of classical and patient-preferred music on anxiety and recovery after ınguinal hernia repair: a prospective randomized controlled study.. Perioper Med (Lond).

[16] Dagher CF, Abboud B, Richa F (2009). Effect of intravenous crystalloid infusion on postoperative nausea and vomiting after thyroidectomy: a prospective, randomized, controlled study.. Eur J Anaesthesiol.

[17] Haentjens LL, Ghoundiwal D, Touhiri K (2009). Does infusion of colloid influence the occurrence of postoperative nausea and vomiting after elective surgery in women?. Anesth Analg.

[18] Dabu-Bondoc S, Vadivelu N, Shimono C (2013). Intravenous dextrose administration reduces postoperative antiemetic rescue treatment requirements and postanesthesia care unit length of stay.. Anesth Analg.

[19] Rao V, Bala I, Jain D, Bharti N (2017). Effect of intravenous dextrose administration on postoperative nausea and vomiting in patients undergoing laparoscopic cholecystectomy: a randomised controlled trial.. Eur J Anaesthesiol.

[20] Mishra A, Pandey RK, Sharma A (2017). Is perioperative administration of 5% dextrose effective in reducing the incidence of PONV in laparoscopic cholecystectomy?: A randomized control trial.. J Clin Anesth.

[21] Agarwal A, Dhiraaj S, Tandon M, Singh PK, Singh U, Pawar S (2005). Evaluation of capsaicin ointment at the Korean hand acupressure point K-D2 for prevention of postoperative nausea and vomiting.. Anaesthesia.

[22] Ljungqvist O, Nygren J, Thorell A (2002). Modulation of post-operative insulin resistance by pre-operative carbohydrate loading.. Proc Nutr Soc.

[23] Feldbauer R, Heinzl MW, Klammer C (2022). Effect of repeated bolus and continuous glucose infusion on a panel of circulating biomarkers in healthy volunteers.. PLoS One.

[24] Oh J, Lee W, Ki S, Suh J, Hwang S, Lee J (2024). Assessment of preoperative anxiety and influencing factors in patients undergoing elective surgery: an observational cross-sectional study.. Medicina (Kaunas).

[25] Uçaner B, Buldanli MZ, Çimen Ş (2024). Investigation of factors that may potentially affect anxiety in patients undergoing esophagogastroduodenoscopy and evaluation of sedation effect.. Medicine (Baltimore).

[26] Hausel J, Nygren J, Lagerkranser M (2001). A carbohydrate-rich drink reduces preoperative discomfort in elective surgery patients.. Anesth Analg.

[27] Mousavie SH, Negahi A, Hosseinpour P, Mohseni M, Movassaghi S (2021). The effect of preoperative oral versus parenteral dextrose supplementation on pain, nausea, and quality of recovery after laparoscopic cholecystectomy.. J Perianesth Nurs.

[28] Mitchell VA, Jeong HJ, Drew GM, Vaughan CW (2011). Cholecystokinin exerts an effect via the endocannabinoid system to inhibit GABAergic transmission in midbrain periaqueductal gray.. Neuropsychopharmacology.

